# Equity impact of participatory learning and action community mobilisation and mHealth interventions to prevent and control type 2 diabetes and intermediate hyperglycaemia in rural Bangladesh: analysis of a cluster randomised controlled trial

**DOI:** 10.1136/jech-2021-217293

**Published:** 2022-03-11

**Authors:** Malini Pires, Sanjit Shaha, Carina King, Joanna Morrison, Tasmin Nahar, Naveed Ahmed, Hannah Maria Jennings, Kohenour Akter, Hassan Haghparast-Bidgoli, A K Azad Khan, Anthony Costello, Abdul Kuddus, Kishwar Azad, Edward Fottrell

**Affiliations:** 1 Institute for Global Health, University College London, London, UK; 2 Centre for Health Research and Implementation, Diabetic Association of Bangladesh, Dhaka, Bangladesh; 3 Department of Global Public Health, Karolinska Institute, Stockholm, Sweden; 4 Department of Health Sciences, University of York, York, UK

**Keywords:** epidemiology, diabetes mellitus, international health

## Abstract

**Background:**

A cluster randomised trial of mHealth and participatory learning and action (PLA) community mobilisation interventions showed that PLA significantly reduced the prevalence of intermediate hyperglycaemia and type 2 diabetes mellitus (T2DM) and the incidence of T2DM among adults in rural Bangladesh; mHealth improved knowledge but showed no effect on glycaemic outcomes. We explore the equity of intervention reach and impact.

**Methods:**

Intervention reach and primary outcomes of intermediate hyperglycaemia and T2DM were assessed through interview surveys and blood fasting glucose and 2-hour oral glucose tolerance tests among population-based samples of adults aged ≥30 years. Age-stratified, gender-stratified and wealth-stratified intervention effects were estimated using random effects logistic regression.

**Results:**

PLA participants were similar to non-participants, though female participants were younger and more likely to be married than female non-participants. Differences including age, education, wealth and marital status were observed between individuals exposed and those not exposed to the mHealth intervention.

PLA reduced the prevalence of T2DM and intermediate hyperglycaemia in all age, gender and wealth strata. Reductions in 2-year incidence of T2DM of at least 51% (0.49, 95% CI 0.26 to 0.92) were observed in all strata except among the oldest and least poor groups. mHealth impact on glycaemic outcomes was observed only among the youngest group, where a 47% reduction in the 2-year incidence of T2DM was observed (0.53, 95% CI 0.28 to 1.00).

**Conclusion:**

Large impacts of PLA across all strata indicate a highly effective and equitable intervention. mHealth may be more suitable for targeting higher risk, younger populations.

**Trial registration number:**

ISRCTN41083256.

**What is already known on the subject**

- The DMagic trial in rural areas of Bangladesh reported the first large-scale, population-level evidence concerning the effectiveness and cost-effectiveness of participatory learning and action (PLA) community mobilisation and mHealth interventions for reducing the prevalence of intermediate hyperglycaemia and type 2 diabetes in the general population and 2-year incidence of type 2 diabetes among individuals identified with intermediate hyperglycaemia. The PLA intervention achieved absolute reductions in prevalence and incidence of 21% and 9%, respectively. The mHealth intervention increased population-level knowledge and awareness of diabetes, but no changes in disease occurrence were observed.

**What this study adds**

- This study investigates the equity of the exposure to and effects of the PLA and mHealth interventions. Results show PLA community mobilisation to be an equitable population-level intervention that can benefit entire communities, including those often excluded from interventions, such as women, older adults and the poorest. Ongoing research is exploring models of national scale-up and adaptation to urban contexts in Bangladesh and elsewhere. mHealth health-promoting interventions may have a role to play in improving health outcomes in certain high-risk groups and should be considered as part of multicomponent, multisectorial responses to diabetes risk.

## Introduction

The prevalence of type 2 diabetes mellitus (T2DM) is growing in South Asia due to a mixture of complex genetic predispositions and increases in obesity and overweight, sedentary behaviour, energy-dense diets and an ageing population.^1 2^ Currently, more educated, more affluent and urban populations in south Asia have a higher prevalence of T2DM, associated with rapid economic development.^3^ However, there is growing evidence of T2DM increasing significantly in low-income rural settings.^1^ Recent estimates of prevalence of T2DM in Bangladesh range from 8% to 12%,^4–7^ and for intermediate hyperglycaemia, defined as abnormal fasting glucose or impaired glucose tolerance, range from 16% to 22%.^7 8^

Health equity is defined as the absence of systematic disparities in health, or its social determinants, between more and less advantaged social groups.^9^ Inequity in health often falls along socioeconomic lines of wealth, age and gender. Other factors that cause changes in health include place of residence (rural/urban); race, ethnicity, culture and language; occupation; religion; and education. Thus, where one is born, lives and works cause health inequities that may be observed across a population in terms of disease, health outcomes and access to health care.^10^

Being poor may cause one to avoid seeking care for diabetes due to high financial costs, resulting in worse health outcomes and possible inability to work, further leading to financial vulnerability.^11^ Gender inequity stemming from differences in power, entitlements and opportunities is an important driver of health outcomes.^12 13^ Women in rural Bangladesh have been shown to have lower access to healthcare compared with men, with reasons including limited autonomy in the choice to seek care, not being allowed to visit healthcare facilities unaccompanied and lower levels of health literacy.^14–16^ Due to higher levels of multimorbidity in older populations, including T2DM, and high out-of-pocket expenditure, healthcare evading behaviour to avoid catastrophic health expenditures has been observed in older adults.^17^

Equity in health and healthcare is central to the Sustainable Development Goals agenda,^18 19^ and so there is urgent need for cost-effective and equitable intervention strategies to prevent and control adverse health outcomes.

The DMagic cluster randomised controlled trial (cRCT) evaluated a participatory learning and action (PLA) community mobilisation intervention and mHealth phone messaging to prevent and control T2DM and intermediate hyperglycaemia in rural communities in Bangladesh.^20^ Online supplemental table 1 shows the baseline characteristics of the populations in each arm. The cRCT showed large, significant reductions in prevalence of T2DM and intermediate hyperglycaemia in PLA intervention clusters compared with control (adjusted OR 0.36, 95% CI 0.27 to 0.48). The 2-year cumulative incidence of T2DM among a cohort of people with intermediate hyperglycaemia was also significantly reduced in PLA clusters compared with control (aOR 0.39, 95% CI 0.24 to 0.65).^20^ There was no evidence of effect in the mHealth arm on population prevalence of intermediate hyperglycaemia and T2DM (aOR 0.93, 95% CI 0.74 to 1.16) or on incidence of T2DM among individuals with intermediate hyperglycaemia (aOR 1.02, 95% CI 0.73 to 1.43). In this study, we analyse the equity of participation and exposure to the two DMagic interventions and their impacts stratified by sociodemographic characteristics.

10.1136/jech-2021-217293.supp1 Supplementary data

## Methods

The DMagic trial took place in 96 villages (approximate population of 125 000) across four purposefully selected subdistricts (‘upazillas’) in Faridpur District, Bangladesh, from June 2015 to June 2018. Villages, which acted as trial clusters, were randomly allocated to either the mHealth, PLA or control arm (32 in each). Men and non-pregnant women aged 30 years and older were the units of analysis.^21^ Full methods for the trial have been published previously.^20 21^

### Setting

Situated on the banks of the Padma River, Faridpur District has a population of over 1.7 million in an area of 2050 km^2^ and has mainly an agricultural economy. The population is primarily Bengali and 90% are Muslim, with the remaining population being Hindu. Faridpur District is divided into nine upazillas (subdistricts), which in turn are divided into unions of approximately 25 000 population.^22^ Primary care is provided by the government at union health and family welfare centres, community clinics and upazilla health complexes. Private and non-governmental institutions that provide healthcare are also available in Faridpur District, including the Diabetic Association Medical College Hospital in Faridpur city. The study was conducted in the upazillas of Boalmari, Saltha, Madhukhali and Nagarkanda. Across the upazillas, 96 villages with a population between 750 and 2500 were selected for inclusion in the study.^21^

### DMagic interventions and randomisation

The PLA intervention was implemented through 18 monthly group meetings focused on T2DM prevention and control. Groups worked through a cycle of problem identification, planning and implementing strategies to address these problems with the wider community, and evaluation. Groups were gender specific, and all members of the community were welcome to attend and participate in the intervention. Communities developed a range of context-specific strategies, including increased opportunities for exercise and access to fresh fruit and vegetables.^23 24^ An equal number of men’s and women’s groups were established within each village, with a total of 122 groups facilitated by 16 facilitators (8 men and 8 women) across the 32 PLA villages. Facilitators were locally recruited people who had completed higher secondary education and 14 days of training about PLA intervention content and structure.

The mHealth intervention involved voice messages promoting behaviour change to reduce diabetes risk.^25^ Messages were sent two times per week over 14 months. The mHealth intervention was available to all individuals with access to a mobile phone (ie, their own or a family member’s) in the mHealth intervention clusters. The intervention was publicised through community engagement and marketing activities, and individuals registered to receive messages free of cost at any time throughout the study period.

The 96 villages were randomly allocated (1:1:1) to the community mobilisation (PLA) intervention, mHealth intervention or control, with each upazilla constituting one stratum. The name of each village was written on pieces of paper which were drawn at random by community leaders and representatives at a public orientation and consent meeting in Faridpur town. The first eight villages per upazilla drawn from the bottle were allocated to arm A, the next eight villages to arm B and the final eight villages to arm C. After all 96 villages had been allocated (32 to each trial arm), each of the three arms were randomly assigned to either the community mobilisation (PLA) intervention, mHealth intervention or the control group by simultaneous drawing of arm letter and intervention allocation from two separate bottles. Because of the nature of the interventions being tested, the intervention team could not be masked to allocation. The data collection team was masked to allocation at the cluster and individual levels during the baseline survey.

### Outcomes

Primary outcomes were (1) the combined prevalence of T2DM and intermediate hyperglycaemia among a random sample of adults aged 30 years and above and residing in study clusters, and (2) 2-year cumulative incidence of T2DM in a cohort of individuals identified with intermediate hyperglycaemia at baseline (ie, prior to any intervention).^21^ Intermediate hyperglycaemia is defined as impaired fasting glucose (fasting plasma glucose of 6.1–6.9 mmol/L and plasma glucose 2-hour postingestion of <7.8 mmol/L) or impaired glucose tolerance (fasting plasma glucose <7.0 mmol/L and 2-hour plasma glucose postingestion of ≥7.8 and <11.1 mmol/L. The criteria for T2DM is having a fasting plasma glucose of ≥7.0 mmol/L or 2-hour plasma glucose postingestion of ≥11.1.^26^

Secondary outcomes included mean diastolic and systolic blood pressures; prevalence of hypertension; hypertension control (among those with known hypertension); mean body mass index; prevalence of overweight and obesity and abdominal obesity (waist to hip ratio >0.9 for men and >0.85 for women); health-related quality of life (using EuroQol-5 Dimensions-3 Level (EQ-5D-3L) score)^27^; physical activity (≥150 min of physical activity per week); mean number of fruits and vegetables consumed per day; and knowledge of the causes, symptoms, complications, prevention and control of T2DM. Additional secondary outcomes among people with T2DM were self-awareness of diabetic status and, among those with known diabetes, prevalence of diabetes control, psychological distress (with the Self-reporting Questionnaire-20 (SRQ-20) screening tool),^28^ and receipt of professional medical treatment or advice for diabetes.^21^

### Data collection

A pre-intervention (baseline) survey to collect data on sociodemographic characteristics and primary and secondary outcomes was carried out among a random sample of 13 684 eligible individuals (permanent resident aged 30 years and older) between January 23 and May 30 2016. Sample size was based on overall trial objectives and is described in detail elsewhere.^21^ A target of 143 households with at least one eligible resident was selected from each village using probability proportional to size sampling, and a single eligible adult was selected from each of the selected households for inclusion in the survey via simple random sampling. The survey included an overnight fasting blood glucose measurement in whole capillary blood obtained by finger prick in the middle or ring finger. All individuals without diagnosed T2DM then received a 75 g glucose load dissolved in 250 mL water. A 2-hour postprandial repeat capillary blood test was done to determine glucose tolerance status and to differentiate between individuals with intermediate hyperglycaemia and those with T2DM. Random (non-fasting) blood glucose tests were conducted if individuals reported a prior diagnosis of T2DM by a medical professional. All data were collected using Open Data Kit on Android devices. The baseline survey identified 2470 individuals with intermediate hyperglycaemia who were then included in our intermediate hyperglycaemia cohort.

Sampling and survey methods were repeated among a new random sample of 13 687 eligible individuals for an end-of-study (postintervention) cross-sectional survey conducted between 16 January and 30 April 2018 to assess intervention effects on the prevalence of intermediate hyperglycaemia and T2DM and secondary outcomes in the general population.

The 2470 individuals identified with intermediate hyperglycaemia in the baseline survey were also followed up in the end-of-study survey to measure incidence of T2DM among this cohort.

### Analysis

#### Intervention exposure

The cross-sectional end-of-study survey was used to describe the reach of the PLA and mHealth interventions by comparing participants who received the intervention to those who did not, within each arm. Exposure to PLA groups was defined as those who self-reported participating in meetings monthly or every 2 months; we reasoned that monthly or bimonthly meeting attendance would give participants enough exposure to benefit directly. Participants who reported attending meetings less frequently were considered as non-participants.

Mobile phones within this context are often a shared resource within households and the mHealth intervention encouraged sharing of message content. We therefore defined exposure to mHealth messages as those who self-reported ever having received a message or knowing someone who had received a message. A sensitivity analysis was also performed where we restricted the definition of exposure to include only those who directly received mHealth messages. Socioeconomic and demographic characteristics between exposed populations and non-exposed populations in the PLA and mHealth arm were described and compared with χ² and Fisher’s exact tests as appropriate.

#### Impact

All individuals who provided blood glucose measurements at the end-of-study survey (including those who only provided a random blood glucose measure on the basis of self-reported diagnosis of T2DM by a medical professional) were included in the analysis of intervention effect on the combined prevalence of T2DM and intermediate hyperglycaemia. In the intermediate hyperglycaemia cohort, all individuals for whom a baseline blood glucose measurement revealed intermediate hyperglycaemia and for whom an end-of-study blood glucose measurement was assessed were included in the analysis.^20^ Intervention effects stratified by gender, age groups and wealth tertiles (derived from principal components analysis of asset ownership) are estimated relative to control using random effects logistic regression adjusted for the clustered study design for the primary outcomes and binary secondary outcomes. For continuous secondary outcomes, mixed-effects linear regression is used. Interpretation of results is based on effect size estimates (regression coefficients) and 95% CIs; however, for each separate outcome, we also indicate statistical significance relative to the Bonferroni corrected p value for multiple hypothesis testing. Taking into account that for each outcome, 18 significance tests were performed, the Bonferroni corrected p value is therefore 0.003. All data were analysed in Stata V.SE15.

### Patient and public involvement

Community representatives were involved from the early stages of the research and engaged through a community orientation meeting. We subsequently established community advisory groups, comprising community leaders and representatives, who were consulted on and influenced intervention and survey activities, considering the burden of the intervention and time required to participate in the research. Community leaders promoted participation in the interventions and surveys, and PLA intervention activities were largely determined by community participants themselves. Trial findings were shared with community members who contributed to the interpretation of the data through a process of participatory analysis described elsewhere.^29^

## Results

At the end-of-study cross-sectional survey, data were collected from 11 454 (83.7%) of 13 687 individuals. In the villages assigned to the control arm, physical measurements (blood glucose, blood pressure and anthropometry) and interview survey data were gathered from 3785 (83%) individuals; 44 (1%) provided only physical measurements, and 1 (<1%) completed only the interview survey. In the mHealth arm, 3797 (83%) completed the physical measurements and interview survey; 15 (<1%) completed physical measurements only; and 5 (<1%) completed the survey only. In the PLA arm, 3786 (83%) completed the physical measurements and interview survey; 12 (<1%) completed the physical measurements only; and 9 (<1%) completed the survey only.^20^

From the intermediate hyperglycaemia cohort identified during the baseline cross-sectional survey (n=2470), 704 (85%) in the control arm, 666 (84%) in the PLA arm and 714 (85%) in the mHealth arm were followed up.^20^ Some of the individuals in the intermediate hyperglycaemia cohort also happened to be randomly selected as part of the end-of-study cross-sectional survey (198 in the control sample, 214 in the PLA sample and 196 in the mHealth sample).

There were more male non-responders than female non-responders (1712 (23%) of 7520 men vs 721 (9%) of 7854 women, p<0·001). Male non-responders were younger than male responders (mean difference 2.0 years, p<0·001), whereas female non-responders were slightly older than female responders (mean difference 3.1 years, p<0·001). Reasons for non-response and loss to follow-up included death, pregnancy, migration and refusal.^20^

### Reach of the interventions

Reach of the interventions was assessed among all individuals who participated in the end-of-study survey from the cross-sectional sample.

Table 1 shows participation in the PLA intervention by demographic characteristics, stratified by gender. Age group distribution did not differ among men who participated in PLA groups compared with men who did not. Among women, the oldest age group (above 60 years) was under-represented in the PLA groups (16.3% participated vs 22.5% did not participate), and female participants were more likely to be younger compared non-participants. Compared with non-participants, group participants were more likely to come from the middle (‘poor’) wealth tertile (34.3% of participants vs 27.4% of non-participants). No differences in education, religion and self-reported diabetes status were observed between participants and non-participants. Female group participants were more likely to be married compared with non-participants (85.2% vs 76.5%), but marital status was not associated with participation among men. Both men and women who participated in groups were more likely to be working compared with non-participants.

**Table 1:** Comparison of sociodemographic and diabetic parameters in participatory learning and action arm group participants and non-participants

		Overall			Men			Women		
		Participants n=2282	Non-participants n=1513	P value	Participants n=1027	Non-participants n=709	P value	Participants n=1255	Non-participants n=709	P value
Gender, n (%)	Male	1027 (45.0)	709 (46.9)	0.261						
	Female	1255 (55.0)	804 (53.1)							
Age (years) n (%)	30–39	739 (32.4)	482 (31.9)	0.044	297 (28.9)	214 (30.2)	0.576	442 (35.2)	268 (33.3)	0.006
	40–49	617 (27.0)	390 (25.8)		248 (24.2)	178 (25.1)		369 (29.4)	212 (29.4)	
	50–59	466 (20.4)	280 (18.5)		227 (22.1)	137 (19.3)		239 (19.0)	143 (17.8)	
	≥60	460 (20.2)	361 (23.9)		255 (24.8)	180 (25.4)		205 (16.3)	181 (22.5)	
Religion, n (%)	Muslim	2065 (90.5)	1378 (91.1)	0.542	923 (89.9)	642 (90.6)	0.642	1142 (91.0)	736 (91.5)	0.669
	Hindu	217 (9.5)	135 (8.9)		104 (10.1)	67 (9.5)		113 (9.0)	68 (8.5)	
Marital status, n (%)	Currently unmarried	205 (9.0)	201(13.3)	<0.001	19 (1.9)	12 (1.7)	0.808	186 (14.8)	189 (23.5)	<0.001
	Currently married	2077 (91.0)	1312 (86.7)		1008 (98.2)	697 (98.3)		1069 (85.2)	615 (76.5)	
Education, n (%)	None	1527 (66.9)	1017 (67.3)	0.053*	649 (63.2)	439 (62.0)	0.345*^†^	878 (70.0)	578 (71.9)	0.190†
	Primary	597 (26.2)	358 (23.72)		274 (26.7)	178 (25.1)		323 (25.7)	180 (22.4)	
	Secondary	129 (5.7)	110 (7.3)		79 (7.7)	68 (9.6)		50 (4.0)	42 (5.2)	
	Tertiary	29 (1.3)	27 (1.8)		25 (2.4)	23 (3.3)		4 (0.3)	4 (0.5)	
Occupation, n (%)	Not working	1304 (57.1)	864 (57.1)	0.036*	87 (8.5)	91 (12.8)	0.003*	1217 (97.0)	773 (96.1)	0.092
	Manual labour	772 (33.8)	477 (31.5)		744 (72.4)	461 (65.0)		28 (2.2)	16 (2.0)	
	Non-manual labour	205 (9.0)	172 (11.4)		195 (19.0)	157 (22.1)		10 (0.8)	15 (1.9)	
Wealth tertiles, n (%)	Most poor	851 (37.3)	656 (43.4)	<0.001	391 (38.1)	319 (45.0)	<0.001	460 (36.7)	337 (41.9)	0.021
	Poor	782 (34.3)	414 (27.4)		388 (37.8)	202 (28.5)		394 (31.4)	212 (26.4)	
	Least poor	649 (28.4)	443 (29.3)		248 (24.2)	188 (26.5)		401 (32.0)	255 (31.7)	
Self-reported diabetes status, n (%)	Not reported	2192 (96.1)	1466 (3.1)	0.176	992 (96.6)	687 (96.9)	0.726	1200 (95.6)	779 (96.9)	0.145
	Reported	90 (3.9)	47 (3.1)		35 (3.4)	22 (3.1)		55 (4.4)	25 (3.1)	

P value from χ² test.

*Missing value for ‘education’ and ‘occupation’.

†P value from Fisher’s exact test.

Table 2 describes sociodemographic parameters among individuals exposed to and those not exposed to the mHealth intervention. Gender distribution was similar between those who were exposed to mHealth messages and those who were not. Significant differences in age for both men and women were observed, with mHealth exposed individuals more likely to be in the 30–39 years age group (32.6% of the exposed group vs 19.6% of the non-exposed group). Differences in exposure by wealth for both men and women were observed, with mHealth exposed individuals more likely to be in the poorest wealth group (33.7% of the exposed group vs 23.1% of the non-exposed group). Overall, individuals exposed to the mHealth intervention were more likely to have completed primary or secondary education (26.5% and 6.2%, respectively) than individuals not exposed (17.1% and 2.8%, respectively). Compared with the non-exposed population, individuals exposed to the mHealth intervention were more likely to be Hindu, and mHealth exposed women were more likely to be married than non-exposed women. Self-reported men with diabetes were more likely to be in the mHealth exposed group (3.8%) compared with the non-exposed (1.0%), but no significant difference was observed for women.

**Table 2:** Sociodemographic, socioeconomic and diabetic parameters among individuals exposed and not exposed to the DMagic mHealth intervention

		Overall			Men			Women		
		Exposed n=3117	Not exposed n=685	P value	Exposed n=1443	Not exposed n=295	P value	Exposed n=1674	Not exposed n=390	P value
Gender, n (%)	Male	1443 (46.3)	295 (43.1)	0.125						
	Female	1674 (53.7)	390 (56.9)							
Age (years), n (%)	30–39	1017 (32.6)	134 (19.6)	<0.001	440 (30.5)	54 (18.3)	<0.001	577 (34.5)	80 (20.5)	<0.001
	40–49	843 (27.1)	168 (24.5)		387 (26.8)	70 (23.7)		456 (27.2)	98 (25.1)	
	50–59	644 (20.7)	161 (23.5)		292 (20.2)	61 (20.7)		352 (21.0)	100 (25.6)	
	≥60	613 (19.7)	222 (32.4)		324 (22.4)	110 (37.3)		289 (17.3)	112 (28.7)	
Religion, n (%)	Muslim	2753 (88.3)	637 (93.0)	0.001	1259 (87.3)	274 (92.9)	0.006	1494 (89.3)	363 (93.1)	0.023
	Hindu	364 (11.7)	48 (7.0)		184 (12.8)	21 (7.1)		180 (10.8)	27 (6.9)	
Marital status, n (%)	Currently unmarried	335 (10.8)	114 (16.6)	<0.001	39 (2.7)	12 (4.1)	0.206	296 (17.7)	102 (26.2)	<0.001
	Currently married	2782 (89.3)	571 (83.4)		1404 (97.3)	283 (95.9)		1378 (82.3)	288 (73.9)	
Education, n (%)	None	2050 (65.8)	546 (79.7)	<0.001*	883 (61.2)	218 (73.9)	<0.001*	1167 (69.7)	328 (84.1)	<0.001*
	Primary	826 (26.5)	117 (17.1)		392 (27.2)	62 (21.0)		434 (25.9)	55 (14.1)	
	Secondary	194 (6.2)	19 (2.8)		132 (9.2)	13 (4.4)		62 (3.7)	6 (1.5)	
	Tertiary	47 (1.5)	3 (0.4)		36 (2.5)	2 (0.7)		11 (0.7)	1 (0.3)	
Occupation, n (%)	Not working	1767 (56.7)	431 (62.9)	<0.001	135 (9.4)	52 (17.6)	<0.001*	1632 (97.5)	379 (97.2)	0.237*
	Manual labour	1044 (32.1)	220 (32.1)		1025 (71.0)	212 (71.9)		19 (1.1)	8 (2.1)	
	Non-manual labour	34 (5.0)	34 (5.0)		283 (19.6)	31 (10.5)		23 (1.4)	3 (0.8)	
Wealth tertiles, n (%)	Most poor	1051 (33.7)	158 (23.1)	<0.001	505 (35.0)	80 (27.1)	0.033	546 (32.6)	78 (20.0)	<0.001
	Poor	1066 (34.2)	252 (36.8)		530 (36.7)	122 (41.4)		536 (32.0)	130 (33.3)	
	Least poor	1000 (32.1)	275 (40.2)		408 (28.3)	93 (31.5)		592 (35.4)	182 (46.7)	
Self-reported diabetes status, n (%)	Not reported	2989 (95.9)	669 (97.7)	0.028	1388 (96.2)	292 (90.0)	0.012*	1601 (95.6)	377 (96.7)	0.402*
	Reported	128 (4.1)	16 (2.3)		55 (3.8%)	3 (1.0)		73 (4.4)	13 (3.3)	

P value from χ² test.

*P value from Fisher’s exact test.

Applying a more restricted definition of exposure to include only individuals who directly received messages showed similar patterns of exposure across sociodemographic groups overall, though a greater proportion of women were classified as unexposed, indicating that women were more likely to be exposed indirectly (online supplemental table S2).

### Impact

PLA resulted in similar large reductions in the combined prevalence of T2DM and intermediate hyperglycaemia among men (aOR 0.42, 95% CI 0.32 to 0.55) and women (aOR 0.33, 95% CI 0.23 to 0.46), across all wealth tertiles (aOR 0.40, 95% CI 0.29 to 0.56 in the poorest; aOR 0.32, 95% CI 0.24 to 0.44 in the middle wealth tertile; and aOR 0.37, 95% CI 0.27 to 0.51 in the least poor) and across all age groups (table 3). Results remained statistically significant after the Bonferroni correction. There was no evidence of an mHealth intervention effect on prevalence of intermediate hyperglycaemia and T2DM in any group.

**Table 3:** Combined prevalence of type 2 diabetes and intermediate hyperglycaemia by trial arms and gender, age group and wealth tertile at the end of the study

Characteristics		End-of-study diabetes and hyperglycaemia cases				
		Control n (%)	mHealth n (%)	PLA n (%)	mHealth aOR (95% CI)*	PLA aOR (95% CI)*
Gender	Male	799 (44.5)	720 (41.6)	455 (26.7)	0.90 (0.71 to 1.13) p=0.347	0.42 (0.32 to 0.55) p<0.001*
	Female	1164 (57.5)	1202 (58.2)	703 (34.3)	1.02 (0.77 to 1.35) p=0.887	0.33 (0.23 to 0.46) p<0.001*
Age groups (years)	30–39	590 (46.7)	516 (44.8)	329 (27.3)	0.91 (0.70 to 1.19) p=0.504	0.33 (0.23 to 0.47) p<0.001*
	40–49	509 (46.5)	512 (50.7)	310 (21.0)	1.06 (0.82 to 1.38) p=0.631	0.43 (0.31 to 0.59) p<0.001*
	50–59	385 (54.8)	445 (55.4)	237 (32.1)	1.03 (0.79 to 1.36) p=0.814	0.37 (0.28 to 0.51) p<0.001*
	≥60	479 (57.9)	449 (53.8)	282 (34.8)	0.87 (0.69 to 1.10) p=0.245	0.38 (0.28 to 0.51) p<0.001*
Wealth tertiles	Most poor	623 (55.0)	661 (54.9)	507 (34.2)	0.98 (0.74 to 1.29) p=0.870	0.40 (0.29 to 0.56) p<0.001*
	Poor	610 (48.9)	636 (48.5)	300 (25.3)	0.95 (0.74 to 1.21) p=0.669	0.32 (0.24 to 0.44) p<0.001*
	Least poor	722 (51.7)	616 (48.6)	348 (32.3)	0.89 (0.68 to 1.16) p=0.389	0.37 (0.27 to 0.51) p<0.001*

*Significant at p=0.003 after Bonferroni correction.

PLA, participatory learning and action.

PLA resulted in reductions in the 2-year cumulative incidence of T2DM by 51% among men (aOR 0.49, 95% CI 0.26 to 0.92) and by 60% among women (aOR 0.40, 95% CI 0.23 to 0.69) (table 4). A consistent reduction in incidence of at least 50% was observed in all age groups, with the exception of those aged 60 years and above, where the effect was attenuated and CIs were wide and included the null effect (0.57, 95% CI 0.27 to 1.20) and the least poor (0.56, 95% CI 0.27 to 1.17). PLA impact was greatest among the poorest group, with a 74% reduction (0.26, 95% CI 0.12 to 0.57). Note however, that 95% CIs overlap across all wealth groups. Based on the Bonferroni correction, the reduction in 2-year cumulative incidence remains statistically significant (p<0.003) only in women and the most poor.

**Table 4:** 2-year cumulative incidence of type 2 diabetes among a cohort with intermediate hyperglycaemia at baseline by gender, age groups and wealth tertiles at end of study

Characteristics		End-of-study 2-year cumulative incidence of diabetes among intermediate hyperglycaemic cohort				
		Control n (%)	mHealth n (%)	PLA n (%)	mHealth aOR (95% CI)*	PLA aOR (95% CI)*
Gender	Male	37 (13.7)	29 (11.9)	16 (7.0)	0.87 (0.50 to 1.51) p=0.618	0.49 (0.26 to 0.92) p=0.026
	Female	89 (20.2)	93 (19.6)	43 (9.9)	0.98 (0.66 to 1.45) p=0.932	0.40 (0.23 to 0.69) p=0.001*
Age groups	30–39	38 (19.9)	19 (10.8)	19 (10.5)	0.53 (0.28 to 1.00) p=0.050	0.35 (0.13 to 0.92) p=0.034
	40–49	34 (17.5)	32 (15.8)	12 (7.4)	0.86 (0.50 to 1.47) p=0.586	0.37 (0.18 to 0.74) p=0.005
	50–59	23 (16.2)	38 (24.4)	10 (7.4)	1.71 (0.88 to 3.30) p=0.111	0.38 (0.17 to 0.84) p=0.017
	≥60	31 (16.8)	33 (18.1)	18 (9.7)	1.14 (0.66 to 1.97) p=0.643	0.57 (0.27 to 1.20) p=0.139
Tertiles	Most poor	50 (23.5)	55 (23.9)	26 (9.4)	1.00 (0.64 to 1.57) p=0.986	0.26 (0.12 to 0.57) p=0.001*
	Poor	33 (14.9)	35 (14.7)	14 (7.8)	0.99 (0.59 to 1.67) p=0.974	0.47 (0.24 to 0.92) p=0.028
	Least poor	43 (15.9)	31 (12.7)	18 (8.8)	0.86 (0.40 to 1.86) p=0.695	0.56 (0.27 to 1.17) p=0.123

*Significant at p=0.003 after Bonferroni correction.

PLA, participatory learning and action.

No significant impacts of the mHealth intervention on cumulative incidence of T2DM were observed. However, a potential intervention effect may be apparent in the youngest (30–39) age group (0.53, 95% CI 0.28 to 1.00; p=0.05).

PLA and mHealth interventions had large positive impacts across all gender, age and wealth groups in relation to knowledge and awareness about the causes, symptoms, complications, prevention and control of diabetes, and, among individuals with diabetes, diabetes control and self-awareness of diabetic status (online supplemental table S3). There was no evidence of effects of either mHealth or PLA on secondary outcomes of blood pressure, overweight and obesity, quality of life and well-being, psychological distress among self-reported diabetics, physical activity, and fruit and vegetable consumption, or diabetic treatment and advice in any gender, age group or wealth group (online supplemental table S3).

## Discussion

Our analysis of the impact of a PLA intervention across age, gender and wealth groups shows that despite socioeconomic differences in participation in PLA groups, the intervention achieved large, significant reductions in occurrence of intermediate hyperglycaemia and T2DM in all gender and wealth groups and in all but the oldest age group in communities where PLA was implemented. Exposure to the mHealth intervention was greater among younger, better educated individuals. Although mHealth intervention effects on primary outcomes were not observed in most groups, indications of a potential effect on 2-year incidence of T2DM among 30–39 year olds with intermediate hyperglycaemia may indicate a role for targeted mHealth interventions in diabetes prevention in high-risk individuals.

While we saw some differences in who participated in PLA groups, impacts were relatively consistent. An explanation for this could be in the way PLA works at the community rather than individual level. There is evidence that non-participants might have been motivated to control and prevent diabetes through interacting with group participants and through the creation of an enabling environment for behaviour change. This helps explain how the benefits of the intervention spread across different groups.^23^ Interventions to improve maternal and newborn health in low-income and middle-income countries (LMICs) found that PLA increases confidence and motivation of group participants as well as non-participants from the community, leading to an increase in healthy behaviours at a population level.^30^ Similarly, our results demonstrate that all sectors of society benefited from the intervention and explain an equitable impact even in the absence of equal participation.

The PLA intervention had an equitable impact in men and women. Meetings held in groups segregated by gender may have made it easier for women and men to participate, with groups organised at times and locations convenient to participants and aligned with cultural norms. Group discussions and larger community meetings enabled men and women to come together to plan actions and may have made household conversations about dietary changes easier. For example, women could cook vegetables and less oily food without being criticised by their husbands, and families grew their own vegetables. Women also felt more able to ask for support to seek healthcare.^23 24 31^

Although participation in PLA meetings was higher in the younger age groups of women, the impact of PLA on combined prevalence of T2DM and intermediate hyperglycaemia was observed in all age groups. However, an impact on cumulative incidence of T2DM among the cohort with intermediate hyperglycaemia was not observed in people above 60 years of age. It has been shown that there can be significant reversal of intermediate hyperglycaemia to normoglycaemia in middle-aged people who undergo behavioural interventions such as increased exercise and dietary restrictions,^32^ but those who are 60 years and above are more likely to progress to T2DM.^33^ It is arguable that if PLA works better to reduce the incidence of T2DM among people under 60 years of age, then this would be the ideal demographic to target in future interventions. Thus, it is important to explore how PLA works among different age groups in future process evaluations.

Although participation in groups was greatest among people in the second wealth tertile (poor), positive impacts of PLA were observed across all socioeconomic strata. This ‘diffusion effect’ of PLA impact across socioeconomic strata was also reported by Houweling and colleagues, although in relation to neonatal health outcomes.^34^ Therefore, even though approximately 40% of people in PLA clusters reported not directly engaging with the intervention, the hypothesised mechanism of widespread community mobilisation underlying PLA may explain the observed positive impact on the community as a whole. Simple and clear interaction about diabetes and the development of local low-cost solutions to individual and community issues may have made it easy for poorer households to implement and respond to them.

Overall, high exposure to the mHealth intervention was observed, with over 80% of individuals in mHealth clusters receiving or knowing someone who received a message. Widespread accessibility and usage of mobile phones throughout the community explains this. Men and women were equally likely overall to be exposed to mHealth, and there was a higher likelihood for both male and female recipients to be younger and more educated. Marital status did not change the likelihood of receiving messages among men but did among women, where married women were significantly more likely to receive messages. This may be explained by mobile phone ownership in Bangladesh being much higher among men, and thus, married women more readily accessing a mobile phone.^35 36^ Women in the poorest wealth tertiles were more likely to receive the intervention. This may be because wealthier women are often subject to restrictions and may not have been able to give their number and register for the intervention. Poor women have less access to the out-of-pocket healthcare system and thus may have been more interested in registering for an intervention that was free.^37^ Despite the large exposure to the mHealth intervention, the passive, information-giving health-promoting nature of the intervention, while effective at raising understanding and awareness of diabetes, did not achieve changes in primary outcomes.

While overall the mHealth intervention did not impact intermediate hyperglycaemia or T2DM prevalence or T2DM incidence, the potential impact on the 2-year cumulative incidence of T2DM among the youngest group with intermediate hyperglycaemia in the mHealth arm is intriguing. One study assessing a medication adherence promotion intervention through mHealth for low-income adults with T2DM showed that older people were less likely to engage in the intervention.^38^ In the USA, the Cell Phone Activities 2012 showed that from the age 50 onwards, mobile phone usage decreased significantly,^39^ a trend which is likely to be reflected globally. Thus, greater familiarity with mobile phone usage among the younger age group could explain the impact of the intervention among this group. However, the theory of the effect of mHealth on younger people needs to be developed. mHealth impact among the 30–39 age intermediate hyperglycaemia group would be important because reductions in risk in this group could have important impacts on disease trajectory. Given the ease of delivery of mHealth and existing evidence of effectiveness in high risk groups in other settings,^40^ further exploring this as a more targeted intervention at scale is warranted.

### Limitations

A potential limitation in our study is that of multiple hypothesis testing, increasing the risk of type I errors in relation to p value cut-offs of ‘statistical significance’. We have therefore applied the Bonferroni adjustment, which applies more stringent criteria of statistical significance. However, we also acknowledge that Bonferroni is widely regarded as a conservative approach to handling multiple testing and it increases the likelihood of type II errors.^41^ Our interpretation of results therefore emphasises effect size estimates and their CIs in relation to scientifically and biologically plausible effects as part of a prespecified equity analysis of trial data. Further limitations of our analysis relate to small numbers in some subgroups, where we may have been underpowered to detect intervention effects.

## Conclusions

PLA community mobilisation for diabetes prevention and control is an effective and equitable population-level intervention. Further research should be conducted to evaluate the effect of PLA in rural areas of other LMICs with a similar high burden of T2DM. PLA should also be adapted and piloted in urban areas in Bangladesh to inform possible country-wide scale-up of the intervention. mHealth health-promoting interventions may have a role to play in improving health outcomes in certain high-risk groups and as part of multicomponent, multisectorial responses to diabetes risk.

## Data Availability

Data are available upon reasonable request. Deidentified data collected for this study and a data dictionary are available from the corresponding author on reasonable request.
