# Supplementary material for: Equity impact of participatory learning and action community mobilisation and mHealth interventions to prevent and control type 2 diabetes and intermediate hyperglycaemia in rural Bangladesh: analysis of a cluster randomised controlled trial
Source: J Epidemiol Community Health. 2022 Mar 11;76(6):586–94. doi: 10.1136/jech-2021-217293 (PMC9118071; doi:10.1136/jech-2021-217293)
Supplement: Supplementary data [file jech-2021-217293supp001.pdf]

Supplementary Material

S1: Sociodemographic characteristics of the DMagic participants at baseline (pre-intervention)

		Control	mHealth	PLA
**Cluster Level**				
**Villages (clusters)**		32	32	32
**Mean village population aged ≥30 years (SD)**		521 (189)	551 (152)	548 (225)
**Mean number of households (SD)**		269 (97)	282 (79)	285 (112)
**Individual Level**				
**Total participants who completed the survey**		4048	4071	4021
**Age, Years**	30-39	13 (34·4%)	132 (32·7%)	138 (34·5%)
	40-49	991 (24·5%)	1068 (26·2%)	992 (24·7%)
	50-59	767 (19·0%)	765 (18·8%)	761 (18·9%)
	60-100	899 (22·2%)	909 (22·3%)	880 (21·9%)
**Gender**	Male	1950 (48·2%)	1845 (45·3%)	1889 (47·0%)
	Female	2098 (51·8%)	2226 (54·7%)	2132 (53·0%)
**Education**	None	2116 (52·3%)	1950 (47·9%)	1905 (47·4%)
	Primary	921 (22·8%)	852 (20·9%)	1004 (25·0%)
	Secondary	989 (24·4%)	1231 (30·2%)	1086 (27·0%)
	Tertiary	22 (0·5%)	38 (0·9%)	26 (0·7%)
**Literacy**	Literate	1455 (35·9%)	1649 (40·5%)	1561 (38·8%)
	Illiterate	2593 (64·1%)	2422 (59·5%)	2460 (61·2%)
**Marital Status**	Not married	520 (12·9%)	497 (12·2%)	491 (12·2%)
	Married	3528 (87·2%)	3,574 (87·8%)	3530 (87·8%)
**Religion**	Muslim	3660 (90·4%)	3674 (90·3%)	3666 (91·2%)
	Other	388 (9·6%)	397 (9·8%)	355 (8·8%)
**Occupation**	Not working	2216 (54·8%)	2323 (57·1%)	2164 (53·8%)
	Manual Labour	1362 (33·7%)	1296 (31·8%)	1376 (34·2%)
	Non-manual labour	468 (11·6%)	452 (11·1%)	481 (12·0%)
**Wealth Tertile**	Most Poor	1401 (34·6%)	1523 (37·4%)	1124 (28·0%)
	Poor	1354 (33·5%)	1351 (33·2%)	1392 (34·6%)
	Least Poor	1293 (31·9%)	1197 (29·4%)	1505 (37·4%)
**Total participants with baseline data for glycaemic status**		4070	4063	4054
**Glycaemic status**	Normal	2860 (70·3%)	2816 (69·3%)	2801 (69·1%)
	Impaired Fasting Glucose	200 (4·9%)	186 (4·6%)	203 (5·0%)
	Impaired Glucose Tolerance	632 (14·7%)	655 (16·1%)	594 (14·7%)
	Diabetes	378 (9·3%)	406 (10·0%)	456 (11·3%)

Pires M, *et al. J Epidemiol Community Health* 2022; 76:586–594. doi: 10.1136/jech-2021-217293

S2: Sociodemographic and diabetic parameters among individuals exposed and not exposed to the DMagic mHealth intervention where exposure is defined as a person having ever received an mHealth message.

		Overall			Men			Women		
		Exposed n=2287	Not exposed n=1470	p-value	Exposed n=1184	Not exposed n=537	p-value	Exposed n=1103	Not exposed n=933	p-value
**Gender**	male	1184 (51·8%)	537 (36·5%)	<0·001						
	female	1103 (48·2%)	933 (63·5%)							
**Age (years)**	30-39	820 (35·9%)	325 (22·1%)	<0·001	395 (33·4%)	97 (18·1%)	<0·001	425 (38·5%)	228 (24·4%)	<0·001
	40-49	655 (28·6%)	350 (23·8%)		334 (28·2%)	121 (22·5%)		321 (29·1%)	229 (24·5%)	
	50-59	438 (19·2%)	351 (23·9%)		229 (19·3%)	118 (22·0%)		209 (19·0%)	233 (25·0%)	
	≥60	374 (16·4%)	444 (30·2%)		226 (19·1%)	201 (37·4%)		148 (13·4%)	243 (26·1%)	
**Religion**	Muslim	2035 (89·0%)	1311 (89·2%)	0·846	1034 (87·3%)	482 (89·8%)	0·150	1001 (88·9%)	829 (88·9%)	0·157
	Hindu	252 (11·0%)	159 (10·8%)		150 (12·7%)	55 (10·2%)		102 (9·3%)	104 (11·2%)	
**Marital Status**	Currently unmarried	174 (7·6%)	264 (18·0%)	p<0·001	24 (2·0%)	25 (4·7%)	0·002	150 (13·6%)	239 (25·6%)	<0·001
	Currently married	2113 (92·4%)	1206 (82·0%)		1160 (98·0%)	512 (95·3%)		953 (86·4%)	694 (74·4%)	
**Education**	None	1401 (61·3%)	1152 (78·4%)	<0·001	689 (58·2%)	395 (73·6%)	<0·001*	749 (64·6%)	757 (81·1%)	<0·001*
	Primary	675 (29·5%)	266 (18·1%)		346 (29·2%)	108 (20·1%)		329 (29·8%)	158 (16·9%)	
	Secondary	173 (7·6%)	40 (2·7%)		120 (10·1%)	25 (4·7%)		53 (4·8%)	15 (1·6%)	
	Tertiary	38 (1·7%)	12 (0·8%)		29 (2·5%)	9 (1·7%)		9 (0·8%)	3 (0·3%)	
**Occupation**	Not working	1160 (50·7%)	1013 (68·9%)	<0·001	90 (7·6%)	96 (17·9%)	<0·001	1070 (97·0%)	917 (98·3%)	0·080
	Manual labour	857 (37·5%)	388 (26·4%)		843 (71·2%)	843 (71·2%)		14 (1·3%)	10 (1·1%)	
	Non-manual labour	370 (11·8%)	69 (4·5%)		251 (21·2%)	251 (21·2%)		19 (1·7%)	6 (0·6%)	
**Wealth Tertiles**	Most poor	822 (35·9%)	387 (26·3%)	<0·001	431 (36·4%)	154 (28·7%)	0·004	391 (35·5%)	233 (25·0%)	<0·001
	Poor	784 (34·3%)	529 (36.0%)		439 (37·1%)	210 (39·1%)		345 (31·3%)	319 (34·2%)	
	Least poor	681 (29·8%)	554 (37·7%)		314 (26·5%)	173 (32·2%)		367 (33·3%)	481 (40·8%)	
**Self-reported diabetes status**	Not reported	2191 (95·8%)	1605 (97·0%)	0·094	1137 (96·0%)	527 (98·1%)	0·024	1054 (95·6%)	897 (96·1%)	0·512
	Reported	96 (4·2%)	49 (3·0%)		47 (4·0%)	10 (1·9%)		49 (4·4%)	36 (3·9%)	

p-value from X^2 test

*p-value from Fisher's Exact test

S3: Impact of mHealth and PLA on secondary outcomes stratified by gender, age groups and wealth tertiles. Measures of effect are beta coefficients where the measure is continuous (eg, mean or median) and are odds ratios where the measure is %.

Secondary Outomes		Gender		Age Group (years)				Wealth Tertiles		
		Male	Female	30-39	40-49	50-59	≥60	1 (most poor)	2	3 (least poor)
Mean diastolic blood pressure (SD) (mmHg)	control	74·27 (10·49)	73·64 (11·76)	72·80 (10·49)	73·49 (11·00)	74·61 (11·60)	75·67 (11·91)	75·34 (11·47)	73·58 (11·21)	73·10 (10·78)
	mHealth	73·06 (10·25)	72·68 (11·25)	71·46 (10·22)	72·84 (10·57)	74·59 (11·47)	73·90 (11·61)	74·29 (11·16)	72·62 (10·70)	71·76 (10·44)
	PLA	74·19 (10·97)	73·42 (11·35)	71·83 (10·25)	74·40 (11·22)	73·83 (10·78)	74·61 (11·60)	74·63 (11·66)	72·72 (10·77)	73·78 (10·86)
Mean diastolic blood pressure coefficient (95%CI)	control	REF	REF	REF	REF	REF	REF	REF	REF	REF
	mHealth	-1·23 (-2·73, 0·27) p=0·109	-1·00 (-2·58, 0·56) p=0·214	-1·21 (-2·81, 0·38) p=0·136	-0·78 (-2·40, 0·83) p=0·340	-0·55 (-2·39, 1·29) p=0·558	-1·70 (-3·53,0·13) p=0·069	-1·00 (-2·81, 0·81) p=0·278	-0·75 (-2·44, 0·95) p=0·388	-0·94 (-6·33, 4·46) p=0·734
	PLA	-0·17 (-1·76, 1·42) p=0·835	-0·34 (-1·97, 1·29) p=0·684	-1·02 (-2·71, 0·65) p=0·229	0·65 (-1·08, 2·38) p=0·463	0·73 (-1·01, 2·48) p=0·410	-1·00 (-2·99, 0·99) p=0·321	-0·65 (-2·43, 1·12) p=0·470	0·68 (-2·60, 1·24) p=0·487	0·29 (-1·34, 1·93) p=0·728
Mean systolic blood pressure (SD) (mmHg)	control	125·32 (19·58)	125·60 (21·85)	120·11 (15·63)	122·43 (18·41)	127·90 (20·19)	127·97 (19·44)	127·17 (20·67)	124·80 (20·50)	125·54 (20·18)
	mHealth	123·37 (17·78)	125·30 (21·68)	118·56 (14·70)	121·67 (17·82)	126·21 (20·52)	126·29 (20·57)	126·83 (21·27)	124·14 (19·47)	122·49 (19·19)
	PLA	125·30 (18·36)	125·33 (20·67)	118·30 (14·60)	124·08 (18·50)	126·51 (20·13)	126·53 (20·19)	126·48 (19·87)	123·65 (18·75)	125·54 (20·18)

**Mean systolic blood pressure coefficient (95%CI)**	**control**	**REF**	**REF**	**REF**	**REF**	**REF**	**REF**	**REF**	**REF**	**REF**
	**mHealth**	-1·90 (-4·42, 0·60) p=0·103	-0·31 (-2·96, 2·33) p=0·816	-1·53 (-3·93, 0·87) p=0·210	-0·80 (-3·44, 1·84) p=0·551	0·01 (-3·25, 3·26) p=0·996	-2·47 (-5·92, 0·97) p=0·160	-0·32 (-3·39, 2·76) p=0·839	-0·130 (-2·76, 2·50) p=0·923	-2·32 (-4·97, -0·34) p=0·087
	**PLA**	-0·02 (-2·50, 2·47) p=0·990	-0·301 (-2·86, 2·26) p=0·818	-1·89 (-4·13, 0·34) p=0·097	1·44 (-1·14, 4·03) p=0·273	1·76 (-1·38, 4·89) p=0·273	-1·55 (-5·43, 2·34) p=0·435	-0·72 (-3·29, 1·85) p=0·584	-0·84 (-3·68, 2·00) p=0·563	0·63 (-2·25, 3·51) p=0·668
**Hypertension n(%)**	**control**	374 (20·8%)	524 (25·9%)	150 (11·9%)	179 (17·4%)	206 (29·3%)	363 (43·9%)	312 (27·5%)	257 (20·6%)	321 (23·0%)
	**mHealth**	302 (17·4%)	551 (26·7%)	122 (10·6%)	175 (17·3%)	218 (27·2%)	338 (40·5%)	333 (27·7%)	267 (20·5%)	251 (19·8%)
	**PLA**	381 (22·3%)	565 (27·6%)	133 (11·0%)	233 (23·3%)	222 (30·0%)	358 (44·2%)	423 (28·6%)	245 (20·7%)	274 (25·4%)
**Hypertension OR (95%CI)**	**control**	**REF**	**REF**	**REF**	**REF**	**REF**	**REF**	**REF**	**REF**	**REF**
	**mHealth**	0·80 (0·62, 1·04) p=0·093	1·04 (0·85, 1·28) p=0·688	0·90 (0·65,1·23) p=0·499	0·99 (0·75,13) p=0·964	0·91 (0·67, 1·23) p= 0·534	0·87 (0·68,1·12) p=0·274	0·99 (0·76,1·29) p=0·945	1·04 (0·80, 1·35) p= 0·787	0·84 (0·67,1·04) p=0·111
	**PLA**	1·08 (0·84, 1·43) p=0·501	1·08 (0·85, 1·36) p=0·543	0·92 (0·65,1·29) p=0·614	1·42 (1·06,1·90) p=0·018	1·06 (0·76, 1·48) p=0·737	1·03 (0·76,1·41) p=0·870	1·02 (0·77, 1·35) p=0·912	1·04 (0·78, 1·38) p=0·789	1·11 (0·84, 1·47) p=0·454
**Hypertension control (among those with known hypertension) n (%)**	**control**	34 (36·2%)	56 (34·4%)	14 (38·9%)	21 (38·9%)	24 (40·7%)	31 (28·7%)	41 (35·7%)	23 (34·3%)	26 (34·7%)
	**mHealth**	36 (39·1%)	85 (38·6%)	14 (50. 0%)	30 (49·2%)	37 (38·5%)	40 (31·5%)	58 (37·9%)	29 (34·9%)	34 (44·7%)
	**PLA**	37 (31·4%)	92 (43·8%)	18 (50·0%)	41 (49·4%)	28 (32·6%)	41 (34·2%)	75 (40·1%)	32 (41·6%)	22 (34·4%)

**Hypertension control (among those with known hypertension) OR (95%CI)**	**control**	**REF**	**REF**	**REF**	**REF**	**REF**	**REF**	**REF**	**REF**	**REF**
	**mHealth**	0·99 (0·51, 1·93) p=0·984	1·16 (0·74, 1·84) p=0·519	1·67 (0·60, 4·62%) p=0·327	1·49 (0·70, 3·17) p=0·303	0·84 (0·36, 1·95) p=0·679	1·04 (0·59, 1·86) p=0·882	1·03 (0·62, 1·71) p=0·916	1·03 (0·52, 2·04) p=0·925	1·34 (0·68, 2·64) p=0·401
	**PLA**	0·71 (0·35, 1·43) p=0·333	1·55 (0·93, 2·57) p=0·090	1·72 (0·48, 6·18) p=0·403	1·55 (0·76, 3·15) p=0·229	0·74 (0·36, 1·53) p=0·422	1·20 (0·66, 2·16) p=0·554	1·10 (0·67, 1·81) p=0·706	1·40 (0·54, 3·61) p=0·486	0·91 (0·44, 1·89) p=0.801
**Mean BMI (SD) (kg/m^2)**	**control**	21·63 (3·27)	22·18 (3·78)	22·75 (3·52)	22·34 (3·62)	21·57 (3·42)	20·45 (3·17)	22·99 (3·75)	21·77 (3·44)	21·18 (3·31)
	**mHealth**	21·43 (3·22)	22·23 (3·92)	22·50 (3·66)	22·25 (3·63)	21·53 (3·61)	20·83 (3·39)	22·89 (3·87)	21·60 (3·35)	21·18 (3·49)
	**PLA**	21·54 (3·23)	22·39 (3·91)	22·53 (3·53)	22·40 (3·64)	21·94 (3·74)	20·81 (3·43)	22·83 (3·88)	21·53 (3·38)	21·39 (3·37)
**Mean BMI coefficient (95%CI)**	**control**	**REF**	**REF**	**REF**	**REF**	**REF**	**REF**	**REF**	**REF**	**REF**
	**mHealth**	-0·19 (-0·46, 0·74) p=0·157	0·05 (-0·25, 0·36) p=0·729	-0·21 (-0·55, 0·13) p=0·223	-0·09 (-0·40, 0·22) p=0·578	-0·02 (-0·46, 0·41) p=0·917	0·35 (0·01, 0·71) p=0·054	-0·51 (-0·36, 0·26) p=0·746	-0·13 (-0·48, 0·22) p=0·464	-0·04 (-0·30, 0·23) p= 0·793
	**PLA**	-0·08 (-0·44, 0·29) p=0·671	0·22 (-0·11, 0·55) p=0·191	-0·22 (-0·60, 0·15) p=0·246	-0·34 (-0·34, 0·41) p=0·858	0·36 (-0·16, 0·87) p=0·174	0·36 (-0·04, 0·77) p=0·080	-0·12 (-0·52, 0·27) p=0·542	-0·20 (-0·58, 0·18) p=0·296	0·14 (-0·23, 0·51) p=0·468
**Overweight or obese n(%)**	**control**	559 (31·1%)	764 (37·8%)	546 (43·2%)	399 (38·8%)	208 (29·6%)	170 (20·6%)	528 (46·6%)	409 (32·8%)	369 (26·4%)
	**mHealth**	480 (27·7%)	782 (37·9%)	458 (39·8%)	372 (36·9%)	244 (30·4%)	188 (22·5%)	544 (45·2%)	394 (30·1%)	322 (25·4%)
	**PLA**	495 (29·0%)	828 (40·4%)	503 (41·7%)	394 (39·4%)	237 (32·1%)	189 (23·3%)	650 (43·9%)	350 (29·5%)	320 (29·7%)

	**control**	**REF**	**REF**	**REF**	**REF**	**REF**	**REF**	**REF**	**REF**	**REF**
**Overweight or obese OR (95%CI)**	**mHealth**	0·85 (0·72, 0·99) p=0·040	1·01 (0·87, 1·17) p=0·927	0·89 (0·74, 1·06) p=0·185	0·93 (0·77, 1·11) p= 0·409	1·02 (0·81, 1·28) p=0·856	1·12 (0·84, 1·49) p=0·456	0·96 (0·81·14) p=0·641	0·9 (0·74, 1·09) p=0·274	0·93 (0·78, 1·11) p=0·403
	**PLA**	0·91 (0·73, 1·13) p=0·398	1·12 (0·96, 1·30) p=0·156	0·95 (0·76, 1·18) p=0·625	1·02 (0·83, 1·25) p=0·876	1·09 (0·84, 1·42) p=0·525	1·20 (0·88,1·63) p=0·250	0·90 (0·74, 1·11) p=0·327	0·85 (0·67, 1·08) p= 0·178	1·1 (0·88, 1·42) p=0·366
	**control**	1450 (80·7%)	465 (23·0%)	327 (25·9%)	281 (27·3%)	195 (27·8%)	205 (24·8%)	319 (28·4%)	286 (23·2%)	394 (28·2%)
**Abdominal Obesity (waist:hip ratio >0.9 for men and >0.85 for women) n(%)**	**mHealth**	1429 (82·5%)	505 (24·46%)	292 (25·4%)	289 (28·6%)	246 (30·6%)	203 (24·3%)	342 (28·4%)	304 (23·2%)	382 (30·2%)
	**PLA**	1428 (83·7%)	455 (22·2%)	314 (26·0%)	316 (31·6%)	217 (29·4%)	205 (25·3%)	433 (29·2%)	301 (25·4%)	315 (29·2%)
	**control**	**REF**	**REF**	**REF**	**REF**	**REF**	**REF**	**REF**	**REF**	**REF**
**Abdominal Obesity OR (95%CI)**	**mHealth**	1·14 (0·85, 1·52) p=0·395	1·05 (0·75, 1·47) p=0·780	0·85 (0·53, 1·36) p=0·503	1·08 (0·70, 1·65) p=0·732	1·03 (0·63, 1·70) p= 0·899	0·97 (0·61, 1·53) p=0·900	0·94 (0·69, 1·28) p=0·680	1·01 (0·67, 1·54) p=0·956	0·99 (0·57, 1·71) p=0·961
	**PLA**	1·22 (0·89, 1·68) p=0·224	0·96 (0·68, 1·35) p=0·815	0·85 (0·53, 1·36) p=0·494	1·24 (0·80, 1·95) p=0·336	1·17 (0·72,1·90) p=0·529	1·18(0·73, 1·93) p=0·499	0·98 (0·66, 1·46) p=0·921	1·13 (0·73, 1·77) p=0·577	1·14 (0·69, 1·88) p=0·608
	**control**	0·85 (0·73, 1.00)	0·73 (0·73, 0·85)	0·85 (0·73, 1·00)	0·85 (0·73, 1·00)	0·73 (0·73, 0·85)	0·73 (0·59, 0·85)	0·85 (0·73, 1.00)	0·85 (0·73, 1.00)	0·73 (0·69, 0·85)
**Median lnEQ5D‡ (IQR)**	**mHealth**	0·85 (0·73, 1.00)	0·73 (0·73, 0·85)	0·85 (0·73, 1·00)	0·85 (0·73, 1·00)	0·80 (0·73, 1.00)	0·73 (0·62, 0·85)	0·85 (0·73, 1.00)	0·85 (0·73, 1.00)	0·80 (0·73, 1.00)
	**PLA**	0·85 (0·73, 1.00)	0·73 (0·73, 0·85)	0·85 (0·73, 1.00)	0·81 (0·73, 1·00)	0·73 (0·73, 0·85)	0·73 (0·59, 0·85)	0·82 (0·73, 1.00)	0·85 (0·73, 1.00)	0·73 (0·73, 0·85)

**Median lnEQ5D‡ coefficient (95%CI)**	**control**	**REF**	**REF**	**REF**	**REF**	**REF**	**REF**	**REF**	**REF**	**REF**
	**mHealth**	0·00 (-0·05, 0·05) p=0·860	0·05 (-0·01 0·11) p=0·078	0·02 (-0·02,0·06) p=0·359	0·04 (-0·01, 0·10) p=0·145	0·05 (-0·01, 0·11) p=0·133	0·02 (-0·07, 0·11) p=0·639	0·00 (-0·06, 0·05) p=0·876	0·03 (-0·03, 0·08) p=0·333	0·05 (0·00, 0·10) p=0.033
	**PLA**	-0·00 (-0·05, 0·04) p=0·906	-0·01 (-0·07, 0·05) p=0·717	-0·01 (-0·05, 0·04) p=0·783	0·00 (-0·05, 0·06) p=0·877	-0·01 (-0·07, 0·57) p=0·817	-0·02 (-0·10, 0·06) p=0·628	-0·02 (-0·07, 0·04) p=0·561	0·00 (-0·05, 0·05) p=0·972	-0·02 (-0·73, 0·02) p=0·321
**Mean self-rated health (SD)**	**control**	77·27 (15·61)	72·08 (16·13)	77·62 (14·34)	76·87 (14·34)	71·9 7 (15·35)	67·51 (17·77)	77·04 (15·03)	75·60 (15·99)	71·51 (16·56)
	**mHealth**	79·82 (15·32)	74·03 (16·06)	79·27 (15·15)	76·89 (15·04)	75·00 (15·08)	70·08 (16·65)	77·40 (15·83)	78·17 (15·79)	74·44 (16·11)
	**PLA**	80·24 (14·51)	76·01 (16·03)	83·51 (13·12)	79·48 (14·72)	78·31 (14·25)	70·24 (15·27)	80·09 (14·92)	78·03 (15·26)	74·89 (16·04)
**Mean self-rated health coefficient (95%CI)**	**control**	**REF**	**REF**	**REF**	**REF**	**REF**	**REF**	**REF**	**REF**	**REF**
	**mHealth**	2·43 (-0·82, 5·69) p=0·143	1·92 (-1·04, 4·89) p=0·204	1·19 (-3·21, 5·61) p=0·595	0·44 (-3·40, 4·27) p=0·824	3·02 (-1·54, 7·58) p=0·195	2·92 (-1·67, 7·51) p=0·213	0·54 (-2·49, 3·58) p=0·725	2·26 (-1·13, 5·64) p=0·191	2·44 (-0·60, 5·37) p=0·115
	**PLA**	2·89 (-0·38, 6·17) p=0·083	3·83 (0·75, 6·93) p=0·015	5·72 (2·63, 8·81) p<0·001	2·50 (-0·93, 5·94) p=0·153	6·34 (1·54,11·13) p=0·009	2·86 (-1·25, 6·97) p=0·172	2·48 (-0·65, 5·60) p=0·120	2·34 (-1·16, 5·85) p=0·190	3·55 (0·76, 6·34) p=0·013
**Median SRQ20 (IQR) among those with self-reported diabetes**	**control**	7.0 (4.0,10.0)	9.0 (7.0,13.0)	7.0 (6.0, 11.0)	9.0 (9.0, 9.0)	12.0 (4.0,20.0)	9·5 (5.0, 14.0)	8.0 (4.0,11.0)	9.0 (8.0,11.0)	10.0 (7.0,14.0)
	**mHealth**	5·5 (4.0,9.0)	7.0 (4.0,9.0)	na	3.0 (0.0,5.0)	8·5 (4.0,11.0)	2.0 (0.0,5·5)	6.0 (3·5,9.0)	5 (3.0,8.0)	9.0 (5.0,11.0)
	**PLA**	8.0 (6.0,11.0)	7·5 (4.0,13.0)	9·5 (8.0,11.0)	4.0 (0.0,15.0)	10·5 (3.0,18.0)	6·5 (0.0,13.0)	7 (4.0,11.0)	9·5 (5.0,13.0)	11.0 (4.0,14.0)

**Median lnSRQ20 coefficient (95%CI)**	**control**	**REF**	**REF**	Insufficient observations for regression analysis				**REF**	**REF**	**REF**
	**mHealth**	-0·12 (-0·43, 0·20) p=0·460	-0·16 (-0·37, 0·05) p=0·141					-0·10 (-0·40, 0·20) p=0·514	-0·51 (-0·80, -0·21) p=0·001	-0·04 (-0·32, 0·24) p=0·797
	**PLA**	0·13 (-0·20, 0·47) p=0·430	-0·04 (-0·28, 0·19) p=0·734					0·15 (-0·08, 0·37) p=0·202	-0·15 (-0·49, 0·19) p=0·377	-0·15 (-0·49, 0·19) p=0·377
**Ability to report ≥1 valid causes of diabetes n (%)**	**control**	1044 (58·1%)	1105 (54·6%)	757 (59·9%)	604 (58·8%)	394 (56·1%)	394 (47·6%)	775 (68·4%)	734 (58·8%)	640 (45·8%)
	**mHealth**	1356 (78·3%)	1610 (78·0%)	956 (83·1%)	841 (83·4%)	622 (77·5%)	547 (65·6%)	1025 (85·1%)	1037 (79·1%)	904 (71·4%)
	**PLA**	1634 (95·7%)	1966 (95·9%)	1170 (96·9%)	967 (96·6%)	714 (96·6%)	749 (92·5%)	1439 (97·2%)	1139 (96·1%)	1022 (94·7%)
**Ability to report ≥1 valid causes of diabetes OR (95%CI)**	**control**	**REF**	**REF**	**REF**	**REF**	**REF**	**REF**	**REF**	**REF**	**REF**
	**mHealth**	3·49 (1·82, 6·71) p<0·001	3·86 (2·19, 6·80) p<0·001	3·64 (2·00, 6·65) p<0·001	4·13 (2·24, 7·59) p<0·001	3·62 (1·98, 6·62) p<0·001	2·63 (1·50, 4·63) p<0·001	3·14 (1·77, 5·55) p<0·001	3·34 (1·81, 6·14) p<0·001	3·68 (1·98, 6·84) p<0·001
	**PLA**	32·96 (15·9, 68·48) p<0·001	42·27 (19·95, 89·56) p<0·001	38·10 (17·35, 83·68) p<0·001	52·67 (20·45, 125·57) p<0·001	40·51 (18·75, 87·53) p<0·001	23·70 (11·67, 48·15) p<0·001	30·61 (14·80, 63·35) p<0·001	26·34 (12·40, 5·96) p<0·001	37·66 (18·12, 78·30) p<0·001
**Ability to report ≥1 valid symptoms of diabetes n (%)**	**control**	1156 (64·3%)	1291 (63·8%)	853 (67·5%)	683 (66·4%)	451 (64·3%)	460 (55·6%)	866 (76·4%)	831 (66·6%)	750 (53·7%)
	**mHealth**	1456 (84·1%)	1734 (84·0%)	1011 (87·8%)	886 (87·8%)	666 (82·9%)	627 (75·2%)	1089 (90·5%)	1102 (84·1%)	999 (78·9%)
	**PLA**	1634 (95·7%)	1979 (96·5%)	1174 (97·3%)	969 (96·8%)	713 (96·5%)	757 (93·5%)	1441 (97·3%)	1143 (96·5%)	1029 (95·4%)

**Ability to report >1 valid symptoms of diabetes OR (95%CI)**	**control**	**REF**	**REF**	**REF**	**REF**	**REF**	**REF**	**REF**	**REF**	**REF**
	**mHealth**	4·28 (1·85, 9·93) p<0·001	4·07 (2·09, 7·90) p<0·001	4·08 (2·01, 8·31) p<0·001	4·60 (2·22, 9·56) p<0·001	3·72 (1·80, 7·67) p<0·001	3·29 (1·62, 6·67) p<0·001	3·43 (1·71, 6·86) p<0·001	3·59 (1·65, 7·80) p<0·001	4·47 (2·06, 9·68) p<0·001
	**PLA**	22·82 (10·12, 51·48) p<0·001	31·85 (13·88, 73·11) p<0·001	28·46 (12·38, 65·53) p<0·001	34·11 (13·26, 87·72) p<0·001	24·92 (10·89, 57·01) p<0·001	19·63 (9·04, 42·06) p<0·001	20·34 (8·86, 46·68) p<0·001	21·12 (8·71, 51·17) p<0·001	29·00 (13·36, 62·96) p<0·001
**Ability to report >1 valid complications of diabetes n (%)**	**control**	1049 (58·4%)	1107 (54·7%)	744 (58·9%)	598 (58·2%)	403 (57·4%)	411 (49·7%)	788 (69·6%)	726 (58·2%)	642 (45·7%)
	**mHealth**	1395 (80·5%)	1674 (81·1%)	972 (84·5%)	866 (85·8%)	641 (79·8%)	590 (70·7%)	1061 (88·1%)	1054 (80·4%)	954 (75·3%)
	**PLA**	1631 (95·6%)	1973 (96·2%)	1172 (97·1%)	964 (96·3%)	714 (96·6%)	754 (93·0%)	1434 (96·8%)	1143 (96·5%)	1027 (95·2%)
**Ability to report >1 valid complications of diabetes OR (95%CI)**	**control**	**REF**	**REF**	**REF**	**REF**	**REF**	**REF**	**REF**	**REF**	**REF**
	**mHealth**	4·92 (2·16, 11·19) p<0·001	5·46 (2·75, 10·85) p<0·001	4·72 (2·26, 9·86) p<0·001	6·37 (3·11, 13·06) p<0·001	4·47 (2·25, 8·85) p<0·001	3·65 (1·85, 7·20) p<0·001	4·23 (2·27, 7·88) p<0·001	4·75 (2·17, 9·65) p<0·001	4·58 (2·17, 9·65) p<0·001
	**PLA**	34·59 (15·93, 74·09) p<0·001	46·26 (21·43, 99·89) p<0·001	47·15 (19·74, 112·64) p<0·001	49·63 (20·25, 121·63) p<0·001	37·60 (18·25, 77·49) p<0·001	25·14 (12·36, 51·14) p<0·001	29·79 (13·95, 63·60) p<0·001	35·61 (15·53, 81·64) p<0·001	45·26 (20·59, 99·45) p<0·001
**Ability to report >1 valid complications of diabetes when prompted n (%)**	**control**	1407 (78·3%)	1519 (75·1%)	991 (78·4%)	808 (78·6%)	542 (77·2%)	585 (70·7%)	983 (86·8%)	978 (78·4%)	965 (69·1%)
	**mHealth**	1523 (87·9%)	1829 (88·6%)	1049 (91·1%)	924 (91·6%)	701 (87·3%)	678 (81·3%)	1131 (93·9%)	1160 (88·5%)	1061 (83·7%)
	**PLA**	1664 (97·5%)	2013 (98·2%)	1191 (98·7%)	982 (98·1%)	725 (88·1%)	779 (96·2%)	1464 (98·9%)	1161 (98·0%)	1052 (97·5%)

**Ability to report >1 valid complications of diabetes when prompted OR (95%CI)**	**control**	**REF**	**REF**	**REF**	**REF**	**REF**	**REF**	**REF**	**REF**	**REF**
	**mHealth**	3·46 (1·23, 9·70) p=0·018	4·26 (1·69, 10·73) p=0·002	3·54 (1·41, 8·87) p=0·007	3·63 (1·40, 9·38) p=0·008	3·09 (1·29, 7·40) p=0·011	2·66 (1·18, 6·00) p=0·018	2·80 (1·16, 6·73) p=0·022	3·17 (1·31, 7·64) p=0·010	3·28 (1·25, 8·67) p=0·016
	**PLA**	16·46 (6·69, 40·44) p<0·001	34·15 (11·74, 99·27) p<0·001	41·59 (12·69, 136·33) p<0·001	22·23 (7·89, 62·69) p<0·001	24·76 (9·81, 62·54) p<0·001	14·47 (6·45, 32·46) p<0·001	18·85 (6·99, 50·82) p<0·001	13·89 (5·71, 33·76) p<0·001	28·07 (10·50, 75·01) p<0·001
**Ability to report >1 valid ways to prevent diabetes n (%)**	**control**	1049 (58·4%)	1107 (54·7%)	904 (71·5%)	720 (70·0%)	486 (69·2%)	511 (61·8%)	892 (78·7%)	889 (71·2%)	840 (60·1%)
	**mHealth**	1395 (80·5%)	1674 (81·1%)	1021 (88·7%)	909 (90·1%)	697 (86·8%)	652 (78·2%)	1106 (91·9%)	1144 (87·3%)	1029 (81·2%)
	**PLA**	1,631 (95·6%)	1,973 (96·3%)	1157 (95·9%)	952 (95·1%)	704 (95·3%)	749 (92·5%)	1410 (95·2%)	1129 (95·3%)	1023 (94·8%)
**Ability to report >1 valid ways to prevent diabetes OR (95%CI)**	**control**	**REF**	**REF**	**REF**	**REF**	**REF**	**REF**	**REF**	**REF**	**REF**
	**mHealth**	4·00 (1·79, 8·92) p=0·001	4·40 (2·25, 8·63) p<0·001	3·78 (1·75, 8·17) p=0·001	5·27 (2·58, 10·74) p<0·001	4·25 (2·07, 8·72) p<0·001	2·96 (1·55, 5·65) p=0·001	3·67 (1·90, 7·10) p<0·001	3·90 (1·82, 8·34) p<0·001	3·85 (1·84, 8·04) p<0·001
	**PLA**	8·62 (4·44, 16·72) p<0·001	15·63 (7·30, 33·47) p<0·001	10·69 (5·37, 21·26) p<0·001	11·77 (5·88, 23·59) p<0·001	11·78 (6·01, 23·10) p<0·001	11·05 (5·68, 21·52) p<0·001	7·97 (3·97, 16·01) p<0·001	8·68 (4·26, 17·70) p<0·001	16·93 (8·70, 32·95) p<0·001
**Ability to report >1 valid ways to control diabetes n (%)**	**control**	1312 (73·0%)	1477 (73·0%)	971 (76·8%)	769 (74·8%)	518 (73·9%)	531 (65·1%)	935 (92·5%)	941 (75·4%)	913 (65·4%)
	**mHealth**	1528 (88·2%)	1811 (87·7%)	1042 (90·5%)	918 (91·0%)	714 (88·9%)	665 (79·7%)	1115 (92·6%)	1167 (89·0%)	1057 (83·4%)
	**PLA**	1620 (94·9%)	1953 (95·3%)	1159 (96·0%)	959 (95·8%)	707 (95·7%)	748 (92·4%)	1414 (95·5%)	1135 (95·8%)	1024 (94·9%)

**Ability to report >1 valid ways to control diabetes OR (95%CI)**	**control**	**REF**	**REF**	**REF**	**REF**	**REF**	**REF**	**REF**	**REF**	**REF**
	**mHealth**	3·68 (1·67, 8·11) p=0·001	3·70 (1·90, 7·23) p<0·001	3·28 (1·58, 6·80) p=0·001	4·29 (2·08, 8·85) p<0·001	4·44 (1·97, 10·05) p<0·001	2·98 (1·59, 5·58) p=0·001	3·08 (1·58, 6·05) p<0·001	3·68 (1·68, 8·06) p<0·001	3·75 (1·74, 8·12) p<0·001
	**PLA**	9·22 (4·42, 19·23) p<0·001	10·25 (4·89, 21·51) p<0·001	8·41 (3·78, 18·70) p<0·001	10·59 (4·98, 22·49) p<0·001	10·68 (4·69, 24·31) p<0·001	9·34 (4·91, 17·75) p<0·001	6·12 (2·79, 13·40) p<0·001	8·62 (4·12, 18·03) p<0·001	14·45 (6·84, 30·52) p<0·001
**Diabetes control among those with known diabetes n (%)**	**control**	13 (43·3%)	20 (35·7%)	5 (50·0%)	4 (20·0%)	8 (33·3%)	16 (50·0%)	14 (33·3%)	9 (52·9%)	10 (37·0%)
	**mHealth**	28 (51·9%)	29 (36·3%)	11 (37·9%)	11 (36·7%)	23 (56·1%)	12 (37·5%)	36 (52·9%)	13 (36·1%)	8 (26·7%)
	**PLA**	35 (66.0%)	33 (47.1%)	5 (50·0%)	18 (47·4%)	21 (65·6%)	24 (55·8%)	40 (55·6%)	19 (59·4%)	9 (47·4%)
**Diabetes control among those with known diabetes OR (95%CI)**	**control**	**REF**	**REF**	**REF**	**REF**	**REF**	**REF**	**REF**	**REF**	**REF**
	**mHealth**	0·96(0·35, 2·62) p=0·940	1·13 (0·34, 3·77) p=0·839	0·58 (0·13, 2·77) p=0·503	1·92 (0·46, 8·01) p=0·369	2·07 (0·44, 9·80) p=0·361	0·43 (0·86, 2·18) p=0·311	2·71 (0·84, 8·83) p=0·096	0·34 (0·05, 2·24) p=0·263	0·50 (0·12, 2·17) p=0·358
	**PLA**	3·04 (0·76, 12·07) p=0·114	1·51 (0·64, 3·55) p=0·349	0·23(0·00, 243·22) p=0·674	3·75 (0·63, 22·25) p=0·146	4·36 (0·96, 19·73) p=0·056	1·17 (0·25, 5·46) p=0·844	3·09 (0·98, 9·73) p=0·054	1·25 (0·31, 4·94) p=0·754	1·29 (0·33, 4·97) p=0.714
**Self-awareness of diabetic status among all those identified as diabetic by objective blood glucose test n (%)**	**control**	37 (21·3%)	57 (18·0%)	11 (9·3%)	24 (17·8%)	25 (25·3%)	34 (24·6%)	45 (27·1%)	18 (13·4%)	31 (16·3%)
	**mHealth**	58 (28·2%)	86 (24·2%)	33 (25·0%)	30 (22·9%)	47 (32·6%)	34 (22·9%)	72 (31·3%)	39 (24·5%)	33 (19·2%)
	**PLA**	57 (46·7%)	80 (44·0%)	14 (24·2%)	43 (46·7%)	35 (56·5%)	45 (48·9%)	78 (49·4%)	34 (44·7%)	25 (35·7%)

**Self-awareness of diabetic status among all those identified as diabetic by objective blood glucose test OR (95%CI)**	**control**	**REF**	**REF**	**REF**	**REF**	**REF**	**REF**	**REF**	**REF**	**REF**
	**mHealth**	1·28 (0·57, 2·86) p=0·550	1·40 (0·90, 2·19) p=0·134	3·42 (1·47, 7·94) p=0·004	1·16 (0·58, 2·33) p=0·667	1·59 (0·73, 3·45) p=0·232	0·85 (0·44, 1·66) p=0·636	1·12 (0·61, 2·04) p=0·724	2·16 (1·05, 4·46) p=0·036	1·26 (0·72, 2·19) p=0·420
	**PLA**	5·27 (2·15, 12·92) p<0·001	4·97 (2·71, 9·15) p<0·001	6·43 (1·38, 30·02) p=0·018	4·73 (2·16, 10·38) p<0·001	6·77 (2·15, 21·38) p=0·001	4·44 (1·88, 10·51) p=0·001	4·36 (1·85, 10·23) p=0·001	7·63 (3·07, 18·96) p<0·001	3·78 (1·70, 8·41) p=0·001
**Receipt of professional treatment or advice for diabetes among those aware of their status n (%)**	**control**	27 (73·0%)	51 (89·5%)	10 (90·9%)	18 (75·0%)	20 (80·0%)	30 (88·2%)	37 (82·2%)	17 (94·4%)	24 (77·4%)
	**mHealth**	49 (84·5%)	73 (84·9%)	25 (75·8%)	28 (93·3%)	40 (85·1%)	29 (85·3%)	63 (87·5%)	31 (79·5%)	28 (84·9%)
	**PLA**	51 (89·5%)	60 (75·0%)	10 (71·4%)	31 (72·1%)	30 (85·7%)	40 (88·9%)	67 (85·9%)	26 (76·5%)	18 (72·0%)
**Receipt of professional treatment or advice for diabetes among those aware of their status OR (95%CI)**	**control**	**REF**	**REF**	**REF**	**REF**	**REF**	**REF**	**REF**	**REF**	**REF**
	**mHealth**	1·65 (0·33, 8·17) p=0·539	0·54 (0·16, 1·90) p=0·341	0·21 (0·01, 6·61) p=0·378	4·64 (0·79, 27·3) p=0·089	0·93 (0·13, 6·82) p=0·947	0·64 (0·14, 2·97) p=0·572	1·27 (0·42, 3·77) p=0·672	0·248 (0·03, 2·28) p=0·218	1·65 (0·43, 6·34) p=0·465
	**PLA**	3·51 (0·56, 22·24) p=0·181	0·36 (0·12, 1·11) p=0·074	1·03 (0.79, 1.35) p=0.830	0·81 (0·25, 2·69) p=0·734	1·71 (0·32, 9·15) p=0·531	0·88 (0·20, 3·91) p=0·863	1·53 (0·37, 6·34) p=0·560	0·178 (0·02, 1·75) p=0·139	0·845 (0·22, 3·21) p=0·805
**Average ≥150 min physical activity per week n (%)**	**control**	1523 (84·8%)	1394 (68·9%)	1030 (81·7%)	858 (83·5%)	538 (76·6%)	488 (59·0%)	888 (78·4%)	973 (78·0%)	1056 (75·6%)
	**mHealth**	1459 (84·2%)	1413 (68·4%)	980 (85·1%)	806 (80·0%)	601 (74·8%)	485 (58·2%)	899 (74·7%)	1028 (78·4%)	945 (74·6%)

	**PLA**	1426 (83·5%)	1385 (67·6%)	966 (80·0%)	796 (79·5%)	578 (78·2%)	471 (58·2%)	1103 (74. 5%)	912 (77·0%)	796 (73·8%)
**Average ≥150 min physical activity per week OR (95%CI)**	**control**	**REF**	**REF**	**REF**	**REF**	**REF**	**REF**	**REF**	**REF**	**REF**
	**mHealth**	0·96 (0·60, 1·54) p=0·863	1·04 (0·60, 1·81) p=0·897	1·23 (1·00,2·57) p= 0·568	1·04 (0·49, 2·21) p=0·926	0·965 (0·51, 1·77) p=0·884	0·97 (0·65, 1·46) p=0·882	0·81 (0·50, 1·30) p=0·375	1·03 (0·64, 1·65) p=0·903	1·10 (0·60, 2·02) p=0·750
	**PLA**	0·91 (0·54, 1·53) p=0·729	0·88 (0·52, 1·51) p=0·644	0·79 (0·40, 1·56) p=0·506	0·79 (0·41, 1·50) p=0·469	0·99 (0·55, 1·83) p=0·998	0·96 (0·64, 1·43) p=0·824	0·74 (0·48, 1·15) p=0·183	0·92 (0·57, 1·47) p=0·719	0·980 (0·54, 1·76) p=0·945
**Mean number of portions of fruit and/or vegetables consumed per day (SD)**	**control**	3·80 (1·79)	3·44 (1·47)	3·78 (1·78)	3·35 (1·44)	3·65 (1·66)	3·22 (1·52)	3·85 (1·73)	3·52 (1·59)	3·49 (1·59)
	**mHealth**	3·77 (1·75)	3·12 (1·45)	3·34 (1·76)	3·36 (1·54)	3·75 (1·71)	3·14 (1·47)	3·64 (1·70)	3·46 (1·54)	3·18 (1·62)
	**PLA**	4·10 (2·16)	3·85 (2·37)	4·14 (2·66)	4·04 (2·80)	3·81 (1·99)	3·39 (1·95)	4·63 (2·62)	3·71 (1·93)	3·33 (1·84)
**Mean number of portions of fruit and/or vegetables consumed per day coefficient (95%CI)**	**control**	**REF**	**REF**	**REF**	**REF**	**REF**	**REF**	**REF**	**REF**	**REF**
	**mHealth**	-0·04 (-0·48, 0·41) p=0·878	-0·32 (-0·69, 0·06) p=0·100	-0·48 (-1·06, 0·78) p=0·091	-0·18 (-0·64, 0·27) p=0·438	-0·08 (-0·36, 0·53) p=0·712	-0·23 (-0·50, 0·40) p=0·818	-0·28 (-0·70, 0·14) p=0·190	-0·13 (-0·52, 0·26) p=0·509	-0·16 (-0·52, 0·20) p=0·382
	**PLA**	0·25 (-0·23, 0·72) p=0·307	0·42 (-0·15, 0·98) p=0·152	0·43 (-0·23, 1·08) p=0·204	0·53 (-0·19, 1·25) p=0·151	0·28 (-0·22, 0·79) p=0·267	0·26 (-0·22, 0·75) p=0·290	0·41 (-0·10, 0·93) p=0·113	0·21 (-0·24, 0·66) p=0·365	0·15 (-0·26, 0·57) p=0·462

‡EuroQol-5D (EQ-5D) using UK tariffs

SRQ-20=Self-Reporting Questionnaire 20-Item

PLA=participatory learning and action

Pires M, *et al. J Epidemiol Community Health* 2022; 76:586–594. doi: 10.1136/jech-2021-217293
